# Distribution and Quantification of Antibiotic Resistant Genes and Bacteria across Agricultural and Non-Agricultural Metagenomes

**DOI:** 10.1371/journal.pone.0048325

**Published:** 2012-11-02

**Authors:** Lisa M. Durso, Daniel N. Miller, Brian J. Wienhold

**Affiliations:** United States Department of Agriculture, Agricultural Research Service, Agroecosystem Management Research Unit, Lincoln, Nebraska, United States of America; U. S. Salinity Lab, United States of America

## Abstract

There is concern that antibiotic resistance can potentially be transferred from animals to humans through the food chain. The relationship between specific antibiotic resistant bacteria and the genes they carry remains to be described. Few details are known about the ecology of antibiotic resistant genes and bacteria in food production systems, or how antibiotic resistance genes in food animals compare to antibiotic resistance genes in other ecosystems. Here we report the distribution of antibiotic resistant genes in publicly available agricultural and non-agricultural metagenomic samples and identify which bacteria are likely to be carrying those genes. Antibiotic resistance, as coded for in the genes used in this study, is a process that was associated with all natural, agricultural, and human-impacted ecosystems examined, with between 0.7 to 4.4% of all classified genes in each habitat coding for resistance to antibiotic and toxic compounds (RATC). Agricultural, human, and coastal-marine metagenomes have characteristic distributions of antibiotic resistance genes, and different bacteria that carry the genes. There is a larger percentage of the total genome associated with antibiotic resistance in gastrointestinal-associated and agricultural metagenomes compared to marine and Antarctic samples. Since antibiotic resistance genes are a natural part of both human-impacted and pristine habitats, presence of these resistance genes in any specific habitat is therefore not sufficient to indicate or determine impact of anthropogenic antibiotic use. We recommend that baseline studies and control samples be taken in order to determine natural background levels of antibiotic resistant bacteria and/or antibiotic resistance genes when investigating the impacts of veterinary use of antibiotics on human health. We raise questions regarding whether the underlying biology of each type of bacteria contributes to the likelihood of transfer via the food chain.

## Introduction

American consumers and producers are increasingly aware of concerns over antibiotic resistance in food. In animal agriculture, the specific concern is that the use of antibiotics in food animals promotes the growth of antibiotic resistant bacteria that can then be transferred to humans via food processing and distribution systems [1]. If these antibiotic resistant bacteria survive the acid pH of the stomach, they can potentially infect humans and cause disease. Additionally, even if the bacteria themselves do not survive outside of the live animal, their antibiotic resistance genes may be available for uptake by other bacteria via a process called horizontal gene transfer (HGT). HGT can occur in bacteria that become associated with a food product that is then ingested by a human, or it can occur directly within the human gastrointestinal tract [2], [3], [4], although the actual rates and frequency of HGT in specific environmental, food associated, and intestinal habitats is not known.

**Figure 1:**
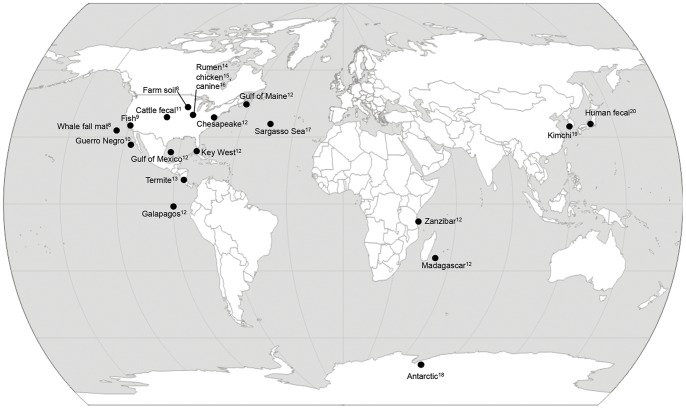
Metagenome samples used in this study. All metagenomes are publicly available on the MG-RAST website. Superscript numbers denote citations listed in the references section. MG-RAST accession numbers and metagenome statistics are listed in Table S1. Metagenome locations were determined using the latitude and longitude metadata linked to each metagenome in MG-RAST.

While a broad conceptual framework has been worked out describing how use of antibiotics in food animals has the potential to adversely impact human health [5], many details regarding the epidemiology and ecology of antibiotic resistance in animal agriculture and food processing systems remain to be described. A number of studies have measured antibiotic resistance in animal production environments [6], [7], and the general public perception is that agricultural environments have more antibiotic resistance than natural and non-agricultural environments. However little empirical data exists to quantify how the levels and types of antibiotic resistance in agricultural settings compare to those in non-agricultural settings. The first step in this comparison is to determine if differences in antibiotic resistance gene distribution can be observed between agricultural and non-agricultural samples, and describe them if present. Here we test the hypothesis that agricultural environments have different antibiotic resistance profiles than non-agricultural environments by quantifying the numbers and kinds of antibiotic resistance genes in publicly available metagenomic samples from 26 environments, including natural and agricultural samples [8]–[20] (**Figure 1**). In addition, we identified bacteria that are likely carrying the antibiotic resistance genes, and determined if there are differences in the diversity or taxonomic distribution of antibiotic resistant bacteria in agricultural and natural settings.

## Methods

### Community Metagenomics

In microbial community metagenomic studies, total DNA is isolated from a complex sample, and then subjected to high-throughput sequencing. Unlike 16S rRNA sequencing which relies on a PCR step to target the 16S rRNA gene specifically, in metagenomic studies there is no PCR step, and the entire genomic DNA of a sample is sheared and sequenced directly. This direct sequencing allows for the collection of quantitative data that is not possible with 16S rRNA-based studies. Metagenomic sequencing theoretically provides information on all of the genes present in a sample, and data analysis tools such as MG-RAST [21] allow for the binning of community genes by function.

**Figure 2:**
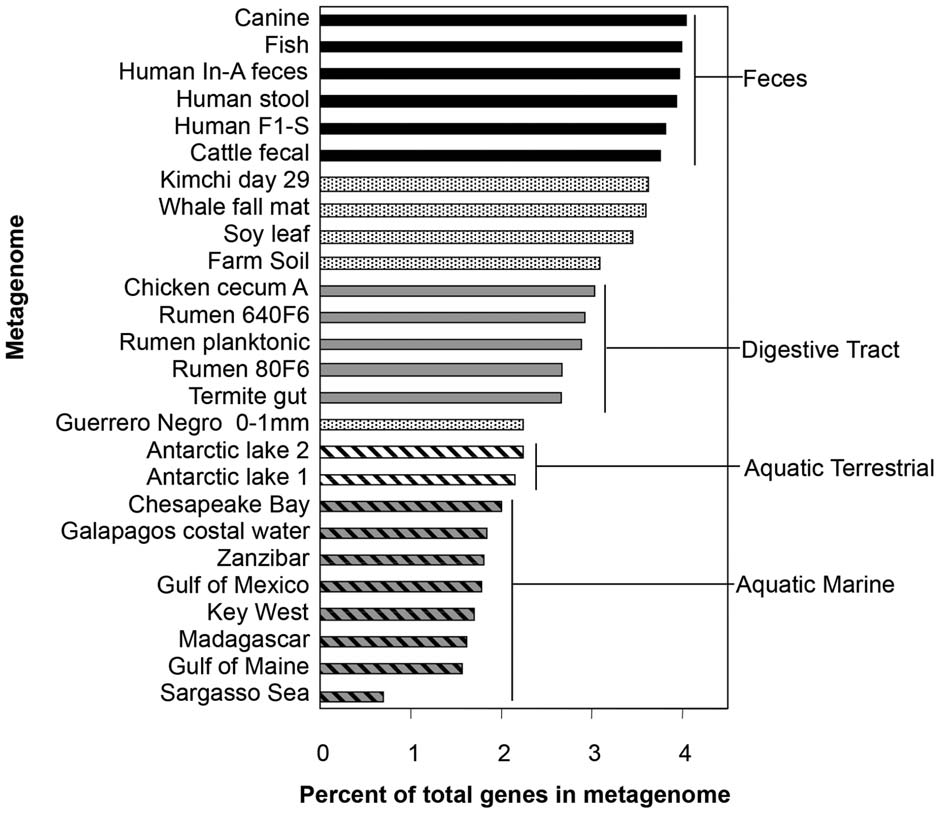
Percent of metagenome coded for by genes responsible for Resistance to antibiotic and toxic compounds. Percent of RATC genes, based on the total number of classified sequences in each metagenome. Metagenomes from microbially dense habitats, such as feces, have a larger proportion of RATC genes compared to metagenomes from habitats that are less microbially dense.

### Measuring Antibiotic Resistance

Antibiotic resistance is a broad term, and is commonly used to denote the presence and/or expression of at least one antibiotic resistance element. There are multiple classes of antibiotics, multiple mechanisms of resistance within classes, and multiple genes within each functional group. In this study, we used the MG-RAST classification of resistance to antibiotic and toxic compounds (RATC) to categorize functional genes associated with antibiotic resistance, and quantified based on absolute numbers or percent within a metagenome or RATC class.

**Figure 3:**
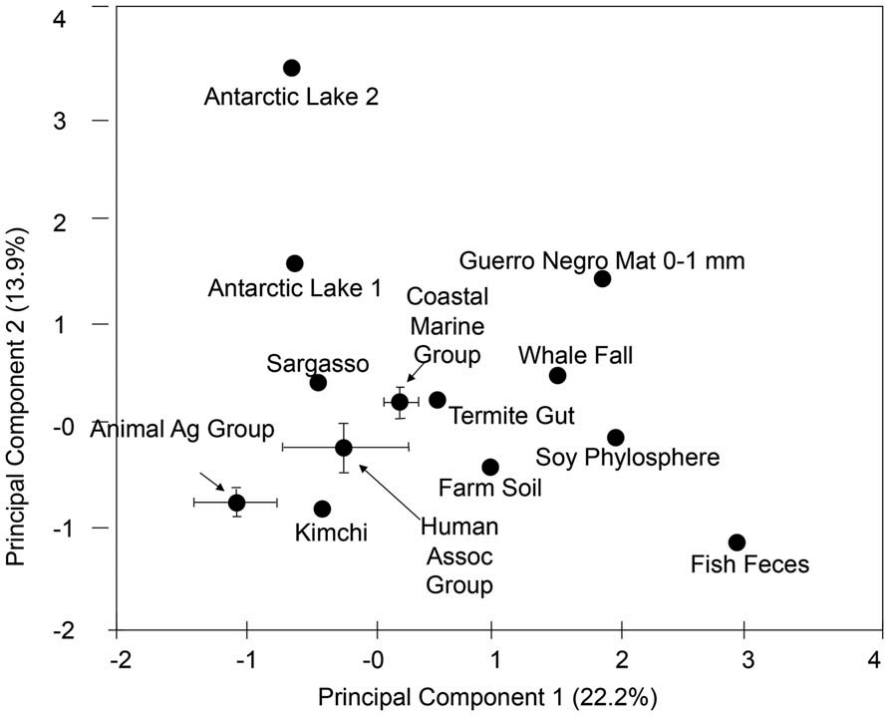
Principal component analysis of 26 metagenomes based on RATC genes. Information regarding resistance to antibiotic and toxic compounds (RATC) was used to perform a principal component analysis of 26 publicly available metagenomes. Both animal agriculture and human associated metagenomes group in quadrant three. Eigenvectors for quadrant three included tetracycline, aminoglycoside adenyltransferase, and multi-drug resistance efflux pump genes.

### Determining Distribution of Functional Genes

In order to determine the distribution of genes associated with resistance to antibiotic and toxic compounds (RATC) from agricultural, environmental, and human-associated samples, we analyzed publicly available metagenomic datasets (**Figure 1**, **Table S1**). Previous work evaluating the distribution of functional genes in metagenomes from nine biomes reported the mean percentage of genes assigned virulence functions was 9.79% [9]. The group of genes we analyzed is the subset of virulence genes that are associated with RATC. The sequences were available through MG-RAST, which was used to annotate the DNA fragments and provide functional assignments [21]. The SEED database [22] was used to link gene function with microbial taxonomy, allowing us to determine which bacteria were likely carrying the antibiotic resistance genes.

**Figure 4:**
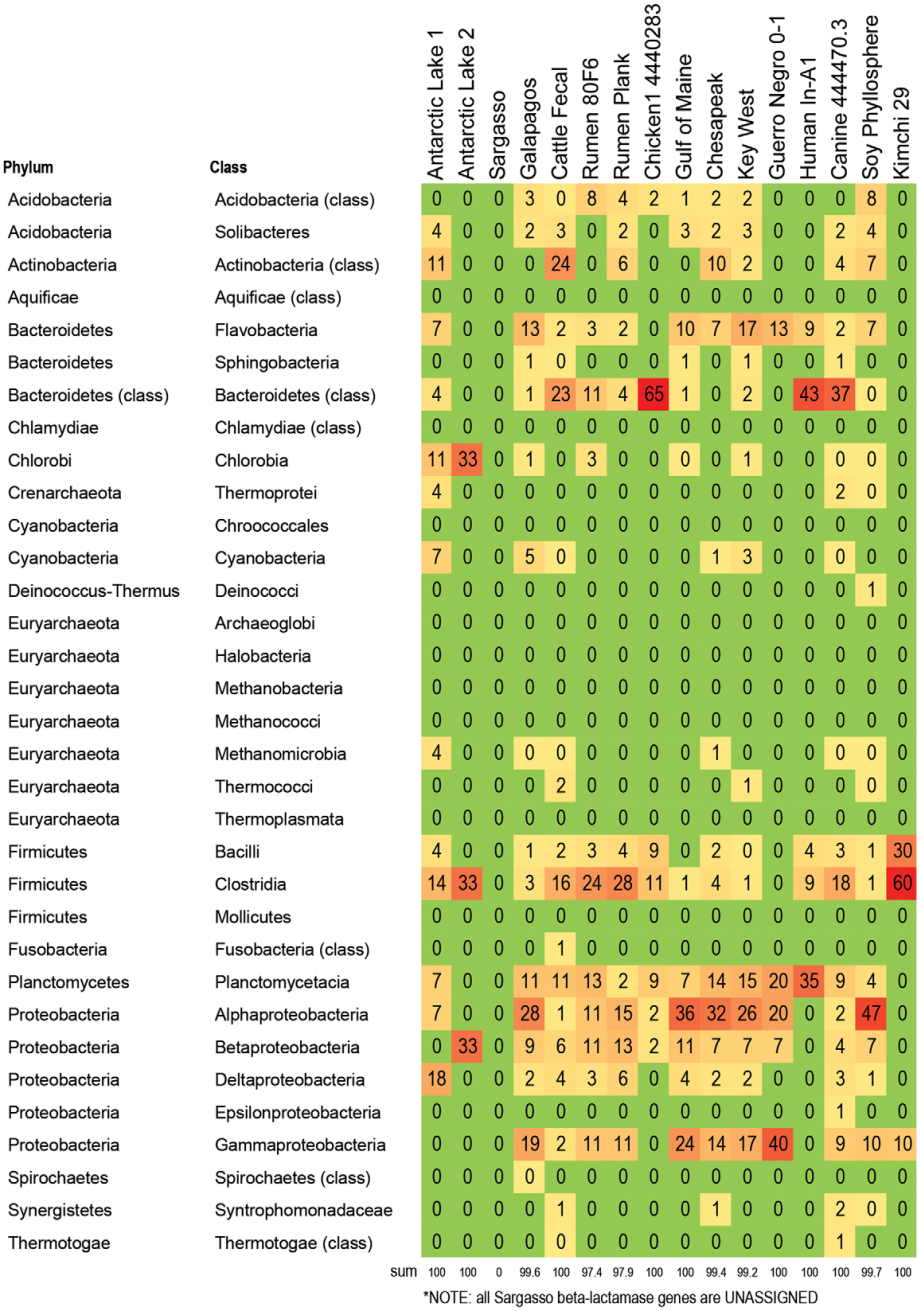
Bacteria responsible for betalactamase genes in 16 metagenomes. Percent of betalactamase genes within each metagenome that are assigned to the listed taxa. For example, 60% of all betalactamase genes in kimchi are associated with Clostridia, 30% of all betalatamase genes in kimchi are associated with Bacilli, and 10% of all betalactamase genes in kimchi are associated with gammaproteobacteria. The alphaproteobacteria carry a larger proportion of the betalactamase genes in marine metagenomes (between 20–36%), compared to metagenomes from food producing animals (1–15%). In contrast, the Clostrida from food producing animals appear to carry proportionally more betalactamse genes than the Clostridia from marine metagenomes. Color formatting indicates low and high values. All true zero values are in green. Values between 0 and 0.5 are listed as 0, but are formatted in yellow. Red indicates the highest value within the chart.

Metagenomes were chosen from those that were publicly available on January 2010. All analyses are based on information provided using MGRAST V2 [21]. The SEED database was used to assign functional classes and taxonomies [22]. In the complete set of MGRAST V2 functional assignments, RATC genes were classified as a subset of virulence genes. MGRAST V2 provided a tabular view of functional assignments where each functional assignment contained a link to the SEED taxonomic assignment. For each metagenome and each RATC category, the tabular view was used to copy the functional assignment and taxonomic link into Excel files. A macro was written to copy the taxonomy line from each SEED taxonomic assignment into the Excel file next to the corresponding functional assignment. The Excel function “Data > text to columns” was used to parse the taxonomic information into individual Excel columns, and the taxonomic data was cleaned and curated by hand. Individual Excel files for each RATC category were combined to generate a single file of linked functional and taxonomic assignments for each metagenome. Principal components analysis of RATC patterns were conducted using the SAS statistical software package version 9.2 (SAS Institute, Cary, NC).

**Figure 5:**
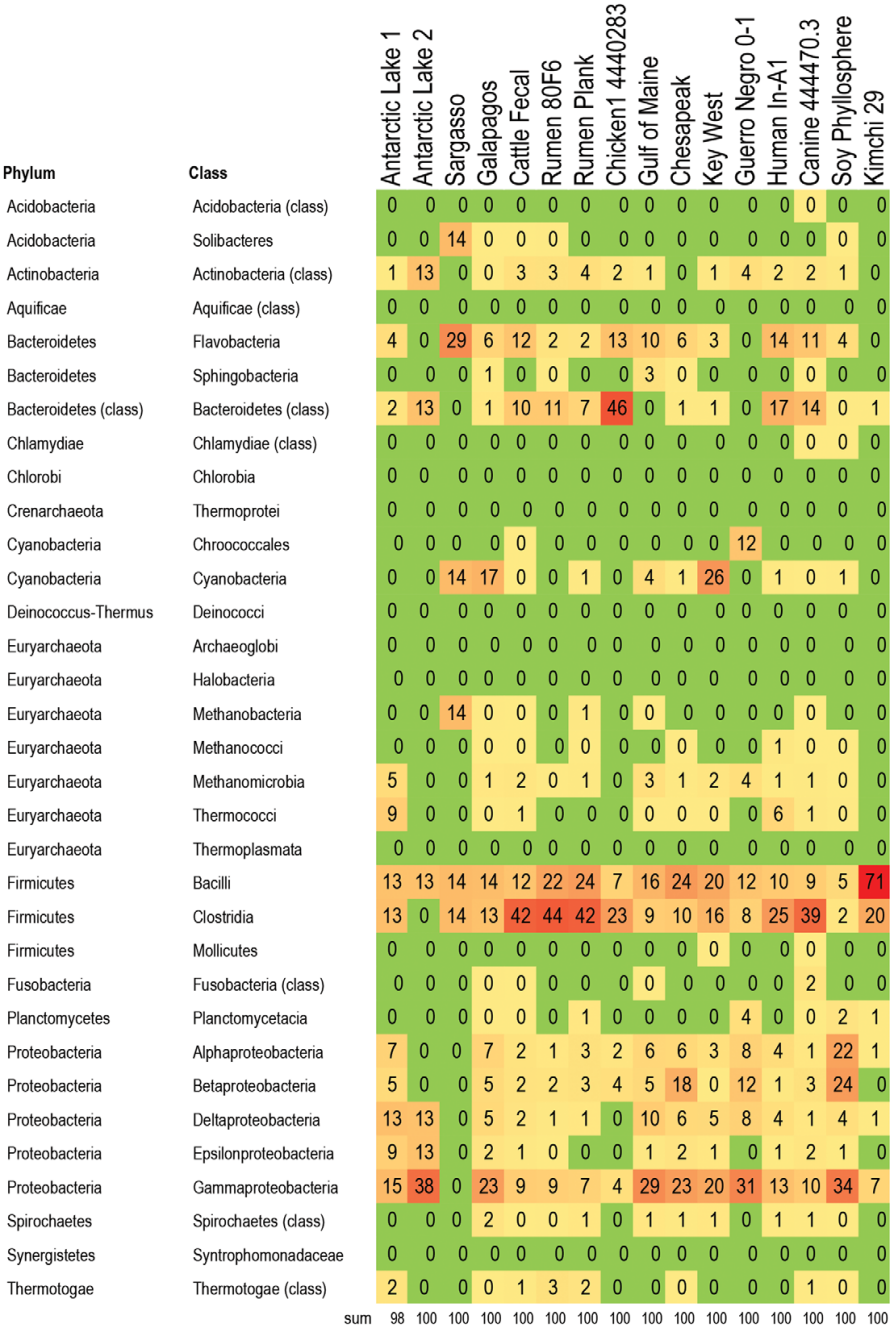
Bacteria responsible for multi-drug resistant efflux pump (MDR efflux) genes in 16 metagenomes. Percent of MDR efflux genes within each metagenome that are assigned to the listed taxa. For example, 71% of the MDR efflux pump genes in kimchi are associated with Bacilli, 20% are associated with Clostridia, and the remaining MDR efflux pump genes are spread across five other bacterial classes. The Clostridia are the main carriers of MDR efflux genes in cattle metagenomes, while the Bacteroidetes are the main carries of MDR efflux genes in the chicken metagenome. Color formatting indicates low and high values. All true zero values are in green. Values between 0 and 0.5 are listed as 0, but are formatted in yellow. Red indicates the highest value within the chart.

## Results and Discussion

### Total Number and Percent of RATC

The RATC group of genes represents a subset of virulence genes that ranged from 0.70% to 4.04% of classified metagenome gene sequences (**Figure 2**). In general, samples from aquatic metagenomes, such as the Gulf of Maine, Chesapeake Bay, Key West, Galapagos and Sargasso Sea, had a lower total number and percentage of assigned fragments associated with RATC, compared with terrestrial metagenomes (**Figure S1**). In other words, microbial communities from marine environments have a smaller percentage of their total genetic resources allocated to RATC genes, suggesting that the bacteria in these habitats encounter fewer antibiotic and toxic compounds, compared to bacteria from the terrestrial and gastrointestinal samples. Based upon prevalence, 22 of the 26 metagenomes could be grouped into one of four classes–fecal (4.04 to 3.75% RATC), gastrointestinal tract (3.03 to 2.66% RATC), aquatic terrestrial ecosystems (2.24 to 2.15% RATC), and aquatic marine ecosystems (2.00 to 0.70%RATC). The percentage RATC in ungrouped metagenomes including kimchi, whale fall, soy phyllosphere, and farm soil ranged from 3.09 to 3.62%. An obvious relationship between increased incidence of RATC and cell density is evident from these samples. Survival in microbially-dense, very active environments (feces, soils, and gastrointestinal tracts) compared to lower density environments (aquatic ecosystems) may involve coping with toxic metabolites and antibiotics. What is the role for anthropogenic antibiotic use and prevalence of RATC? Certainly samples within fecal and gastrointestinal tract groups were likely exposed to anthropogenic antibiotics at some point in time, but the high rate of RATC in kimchi, whale fall, and termite gut, which probably were never treated with antibiotics, indicate that the effect of antibiotic exposure on percent RATC is more complicated.

**Figure 6:**
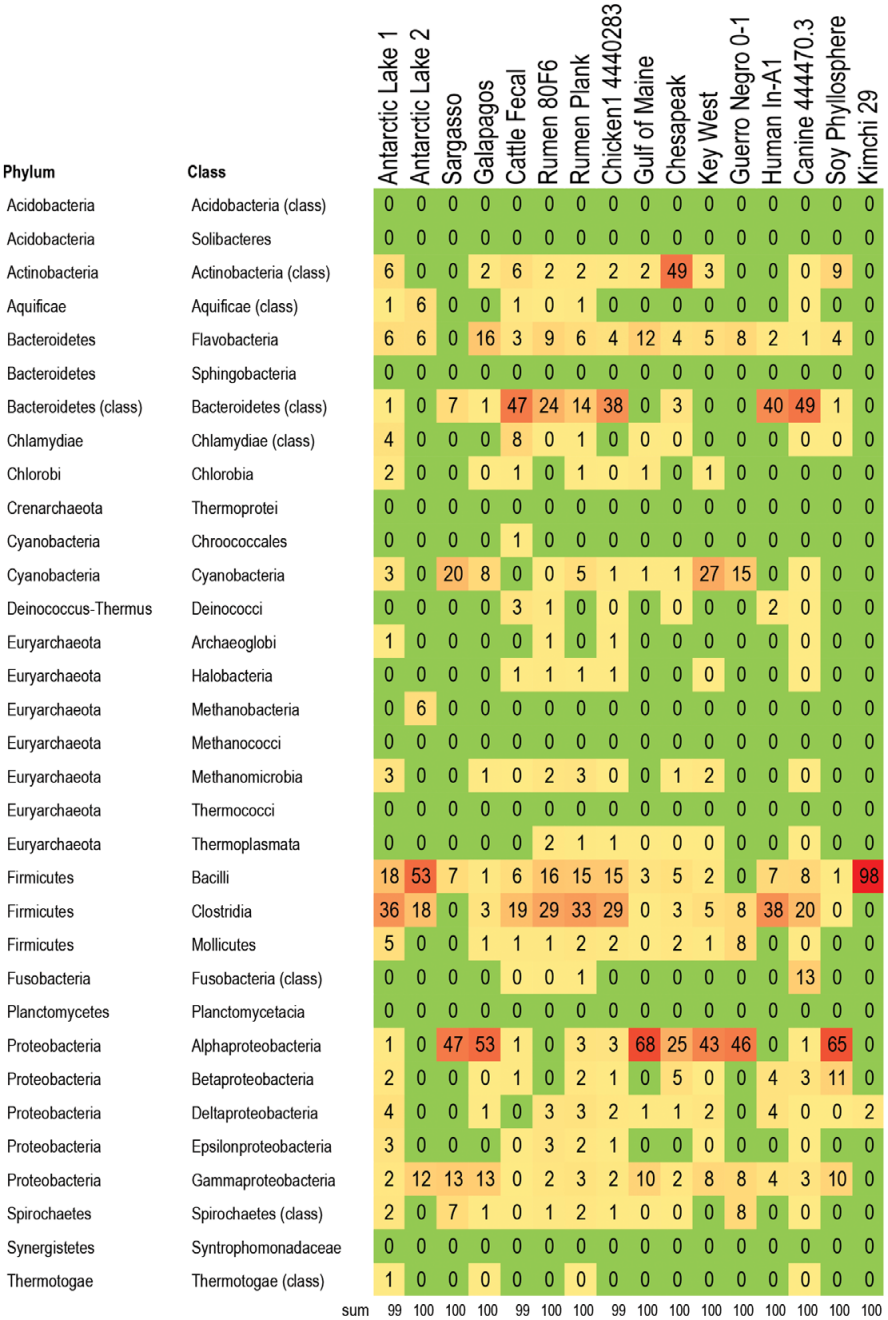
Bacteria responsible for fluoroquinolone resistance genes in 16 metagenomes. Percent of fluoroquinolone resistance genes within each metagenome that are assigned to the listed taxa. For example, almost all (98%) of the fluoroquinolone resistance genes in the kimchi metagenome were associated with Bacilli. The fluoroquinolone resistance genes in food animal metagenomes were found mainly in Clostridia and Bacteroidetes, while the fluoroquinolone resistance genes in marine metagenomes were found primarily in Alphaproteobacteria. The two Antarctic Lake metagenomes also had a high proportion of their fluoroquinolone resistance genes assigned to Clostridia, but unlike the food animal metagenomes, there were very few fluoroquinolone resistant Bacteroidetes. Color formatting indicates low and high values. All true zero values are in green. Values between 0 and 0.5 are listed as 0, but are formatted in yellow. Red indicates the highest value within the chart.

The current metagenomic analysis affords a first glimpse of antibiotic gene distribution across metagenomes. For example, although the four North American costal samples display the same general distribution of RATC genes, slight differences can be observed for specific RATC classes and locations. The Chesapeake Bay metagenome has a higher proportion of assignments in the tripartite Gram negative multi-drug resistance (MDR) (2.6%) and tetracycline resistance (2.6%) categories compared to the Gulf of Main, Key West, and the Gulf of Mexico (range 0.8–1.9% for Gram negative MDR, 0–0.24% for tetracycline), and a lower proportion of the acriflavin resistance cluster and beta-lactamase resistance (13.9% for acriflavin resistance genes in Chesapeake compared to 16.6–21.0% for the others; 9.9% for tetracycline resistance in Chesapeake compared to 15.6–17.5% for the others). Difference in RATC distribution can also be observed in the cattle rumen and fecal samples, with both fluoroquinolone resistance and tetracycline resistance genes being lower in feces (20% for fluoroquinolone, 5% for tetracycline) than in rumen samples (average of 30% for fluoroquinolone, 19% for tetracycline). The differences between the fecal and rumen samples likely reflect the underlying biological functions of the two gastrointestinal locations, and serve as a reminder of the dynamic nature of the ecology of antibiotic resistance genes in food production settings. Two other metagenomes of interest are the fish gut and the fermented kimchi. The fish gut contains a much higher proportion of mercury resistance, mercuric reductase, and cobalt/zinc/cadmium genes than any other metagenome in the analysis (1.65%, 2.26% and 53% respectively for each category in fish, compared to an average of 0.03%, 0.3%, and 16%, respectively, for the other metagenomes studied). The day 29 kimchi fermentation contains proportionately more of the two-protein Gram positive MDR genes (12.3%) compared to any other metagenome in the analysis (next highest value is a human stool at 0.93%). Both the fish and the kimchi results make sense in light of what we already know about the biology of the two systems: fish are known to concentrate mercury, and vegetable fermentations specifically select for Gram-positive organisms. The data set assembled for the current analysis contains three human fecal and three cattle rumen metagenomes. Within each “replicated” metagenome type, the RATC values are fairly similar. While all of the metagenome results await confirmation via replication over multiple samples and over time, the limited data available at this point suggests that there are characteristic RATC profiles for specific metagenomes. Previous work examining metagenomic profiles revealed biome-associated metabolic profiles, including gene assignments to the functional category of “virulence”. The current study extends the conclusions of Disndale et al. [9] to include RATC, one of many subsets in the virulence category.

**Figure 7:**
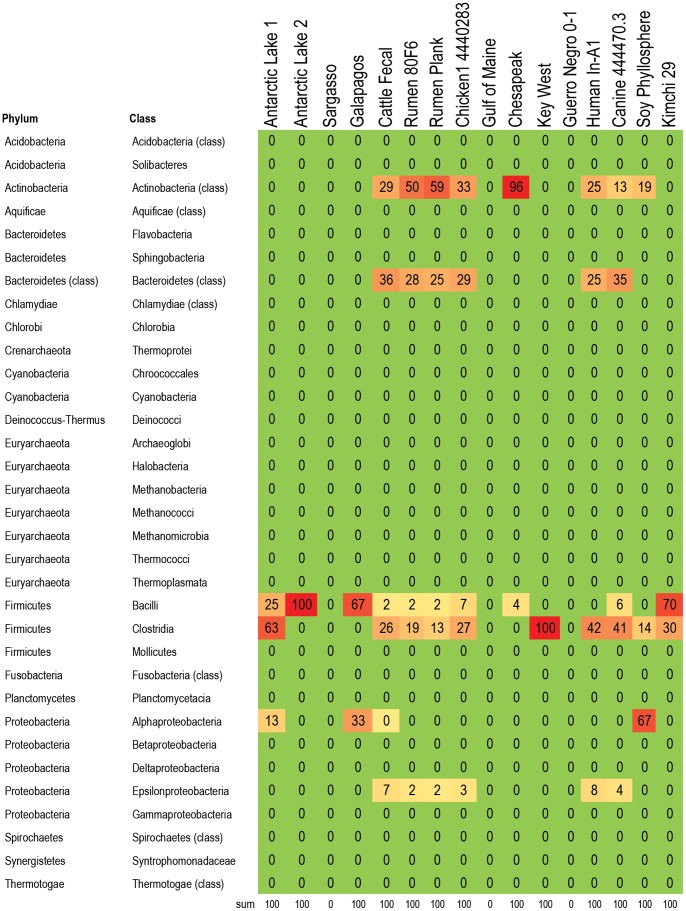
Bacteria responsible for tetracycline resistance genes in 16 metagenomes. Percent of tetracycline resistance genes within each metagenome that are assigned to the listed taxa. For example, 70% of all tetracycline resistance genes in the kimchi metagenome were assigned to Bacilli. Tetracycline genes associated with Bacteroidetes are fairly common in food animal and human associated metagenomes, but were not found in any of the marine or environmental metagenomes examined in this study. A similar pattern was seen for tetracycline resistance genes associated with Epsilonproteobacteria, though the overall percent of tetracycline resistance genes associated with this group was much lower. Color formatting indicates low and high values. All true zero values are in green. Values between 0 and 0.5 are listed as 0, but are formatted in yellow. Red indicates the highest value within the chart.

All metagenomes examined contained antibiotic resistance genes. Thirty-three different RATC categories were represented in the 26 metagenomes examined (**Figure S1**). Of these RATC categories, beta-lactamase resistance, MDR efflux pumps, fluoroquinolone resistance, cobalt/zinc/cadmium resistance, and acriflavin resistance genes were present in all 26 metagenomes. This broad distribution across agricultural, environmental, and human-associated samples indicates that these mechanisms of antibiotic resistance are functionally important in many habitats, however the current analysis is unable to determine if these genes have been transferred between habitats.

**Figure 8:**
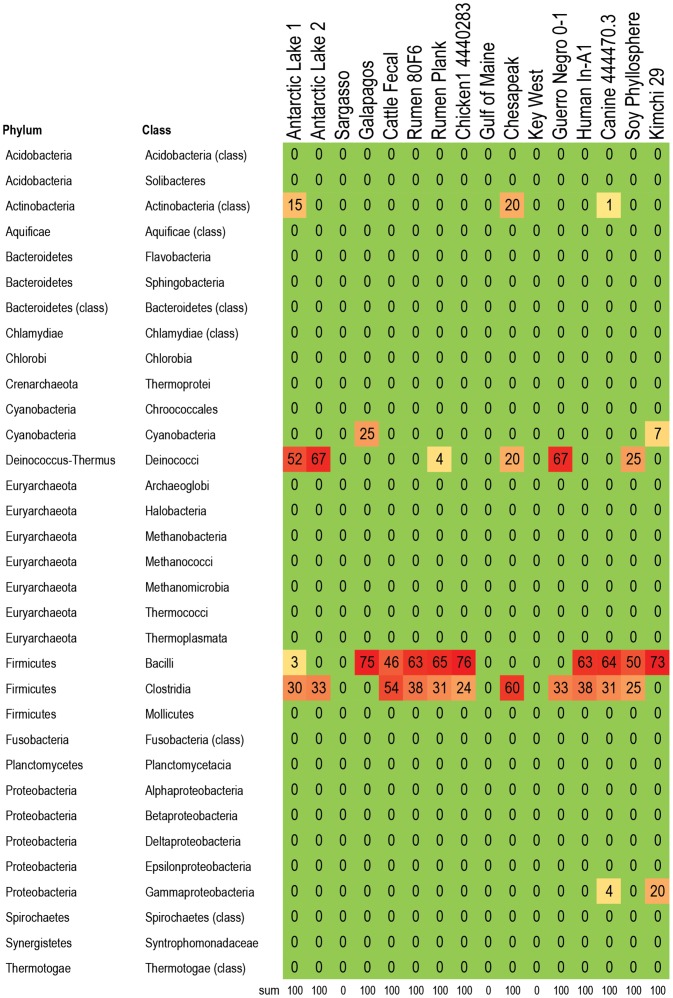
Bacteria responsible for vancomycin resistance genes in 16 metagenomes. Percent of vancomycin resistance genes within each metagenome that are assigned to the listed taxa. For example, 73% of the vancomycin resistance genes in kimchi were attributed to Bacilli. As expected, the vancomycin resistance genes are carried predominantly by members of the phylum Firmicutes. Color formatting indicates low and high values. All true zero values are in green. Values between 0 and 0.5 are listed as 0, but are formatted in yellow. Red indicates the highest value within the cart.

The canine and soy metagenomes were the most diverse in regards to the 33 total RATC categories represented, with 31 and 30 observed RATC categories, respectively, followed by farm soil, human stool and cattle feces with 27 RATC categories observed, and Chesapeake Bay with 25 RATC categories observed. The least diverse metagenome was the Sargasso Sea, with only 7 RATC categories represented.

Principal component analysis based on the number of genes in each of the RATC classes reveals that agricultural, human-associated, and costal marine samples each form distinct groups (**Figure 3** **)**, suggesting that there are differences in the numbers and kinds of antibiotic resistance in these general categories of metagenomes**.** Specifically, agricultural and human-associated samples each form distinct clusters within quadrant III, and aquatic samples, including a coastal marine cluster, were distributed within quadrant IV and nearby in quadrant I (**Figure 3**). Comparing the general distribution of various RATC metagenomes within the plot indicates that the variation between human-and animal agriculture is not disproportionate compared to other systems. For instance the separation of two Antarctic Lakes is roughly the same magnitude separating coastal marine metagenomes from the animal agriculture group. Examination of the Eigen vectors associated with RATC classes indicates that tetracycline, aminoglycoside adenyltransferase, and MDR efflux pump genes were strong determining factors for quadrant III groups. Whether or not these particular RATC classes can serve to distinguish larger numbers of human and agricultural from aquatic marine samples remains to be determined.

### Antibiotic Resistance in Specific Habitats

A subset of 16 metagenomes were examined to determine which bacteria were likely hosts within each metagenome for RATC genes, with a focus on genes associated with common antibiotic resistance classes, and classes that are important in human and veterinary health settings. The five classes chosen were: beta-lactamase resistance, MDR efflux pumps, fluoroquinolone resistance, tetracycline resistance, and vancomycin resistance (**Figures 4** **,** **5** **,** **6** **,** **7** **,** **8**; **Table S2)**: Results indicate that specific RATC gene categories are non-randomly distributed among bacterial taxa. The beta-lactamase resistance, MDR efflux pumps, and fluoroquinolone resistance genes were broadly distributed across 15, 12, and 13 bacterial phyla, respectively, compared to four for tetracycline and five for vancomycin resistance. The broader taxonomic distribution of beta-lactamase, MDR efflux and fluoroquinolone resistance genes suggests that there is a greater diversity of bacteria that are capable of taking up these genes via HGT in natural settings, compared to tetracycline and vancomycin resistance genes. Since little is known about the commensal flora ingested with food, it is unknown whether or not the taxonomic range of the bacteria carrying antibiotic resistance genes may impact human health. Also, HGT rates between various groups of bacteria are not well characterized. When looking at the impact of veterinary antibiotic use on human health, are some kinds of resistance more likely to be transferred between commensal bacteria of different phyla via HGT than others? Are some bacteria phyla more likely than others to horizontally transfer antibiotic resistance genes to particular foodborne pathogens?

### Distribution and Taxonomy of Beta-lactamase Resistance Genes

Beta-lactam antibiotics (**Figure 4**) are widely used in human clinical medicine, and include drugs such as penicillins and cephalosporins. Beta-lactamase genes associated with the human fecal sample accounted for 1% of the total beta-lactamase genes in the 16 metagenomes studied (**Table S3**). The majority of beta-lactamase resistance genes in this particular set were associated with the soy phyllosphere (23%), followed by Galapagos (19%), canine (17%), cattle fecal (10%), and Key West (9%). Across the 16 metagenomes, Beta-lactamase genes were most frequently associated with Alpha- and Gammaproteobacteria, which together account for 36% of all Beta-lactamase genes in the 16 metagenomes studied. It is not clear why the leaf of a soybean plant contained the largest proportion of beta-lactamase genes from the 16 metagenomes examined; however extended-spectrum beta-lactamase gene sequences have been identified on both conventional and organic retail spinach [23]. Since there is no metadata associated with this sample in the database, it is unknown whether or not the source plant was genetically modified. In regards to beta-lactamase genes in the marine Galapagos sample, previous studies have shown low numbers of beta-lactamase resistant enterobacteria in the guts of terrestrial Galapagos (Santa Fe) iguanas [24]. In the marine sample analyzed as part of this study, beta-lactamase genes were distributed across 16 different classes, and the Gammaproteobacteria (class that includes the enterobacteria) accounted for only 19% of all beta-lactamase genes in the metagenome.

### Distribution and Taxonomy of MDR Efflux Pump Genes

MDR efflux pumps (**Figure 5**) are another category of antibiotic resistance genes with relevance for human clinical medicine. The Antarctic and marine-based metagenomes had the overall lowest percentage of RATC genes assigned to MDR efflux pumps, (17.6%), followed by soil (21.5%), cattle rumen (28.6), and human and animal feces (40.4%). The MDR efflux genes were most frequently assigned to Clostridia in the animal agriculture samples and Gammaproteobacteria in the coastal marine samples.

### Distribution and Taxonomy of Fluoroquinolone Resistance Genes

Fluoroquinolone resistance (**Figure 6**) has been of particular interest in the discussion regarding the use of antibiotics in animal agriculture [25]. There were no statistically significant differences in the percent of RATC genes coding for fluoroquinolone resistance genes between the four animal agriculture samples, and the five marine-associated samples. The DNA gyrase subunit B (EC 5.99.1.3) gene was most frequently associated with Clostridia and Bacteroidetes (average 33% of all DNA gyrase subunit B assignments in each metagenome) in fecal metagenomes (cattle, chicken, canine, human), and with Proteobacteria (average 64% of assignments in each metagenome) in marine-associated metagenomes (Gulf of Maine, Chesapeake, Galapagos, Key West, Sargasso). Topoisomerase IV subunit A (EC 5.99.1.-) was most frequently associated with Bacteroidetes in fecal metagenomes (average 76% of all Topoisomerase IV subunit A genes within each metagenome), with Actinobacteria from the Chesapeake Bay metagenome (average 89% of all Topoisomerase IV subunit A genes within each metagenome), and with the Proteobacteria (average 79% of all Topoisomerase IV subunit A genes within each metagenome) from the other coastal marine-associated metagenomes.

### Distribution and Taxonomy of Tetracycline Resistance Genes

Overall, 90% of Tetracycline resistance genes from the 16 metagenomes sampled were associated with canine and animal agriculture metagenomes (**Figure 7**). The human metagenome contained 1.3% of the tetracycline resistance genes from this study, distributed across five gene categories. Tetracycline resistance is very common among soil bacteria, but soil samples were not included in this particular analysis. The only tetracycline resistance gene found in marine-associated metagenomes was Translation Elongation Factor G, which was present in 4.5% of the Chesapeake Bay samples, and less than 1% of the Key West and Galapagos samples. The Gulf of Maine and Sargasso Sea metagenomes did not have any tetracycline resistance gene fragments that could be classified. Marine-associated tetracycline resistance was predominantly found in Actinobacteria (87%), whereas the animal agriculture tetracycline resistance genes were carried by a broader range of bacteria, including Actinobacteria (40%), Bacteroidetes (29%), and Clostridia (21%). In the canine metagenome, 40% of the tetracycline resistance genes were Clostridia, 35% were Bacteroidetes, and only 13% were Actinobacteria.

### Distribution and Taxonomy of Vancomycin Resistance Genes

The canine metagenome accounted for 34% of all vancomycin resistance genes found in the 16 metagenomes examined (**Figure 8**). The majority of vancomycin resistance genes were associated with Bacilli, though there was a single Antarctic Ace Lake metagenome that had a majority of vancomycin resistance genes assigned to Deinococci. The human metagenome accounted for 3% of the total vancomycin resistance genes in the set, with genes assigned to Bacilli and Clostridia. These same two groups were also associated with carriage of the vancomycin resistance genes in the animal agriculture and soy phyllosphere metagenomes.

### Application of Antibiotic Resistance Information to Food Safety

As with all gene-based surveys, the data indicate only the presence or absence of the target gene, but provide no information on whether the gene came from a living or dead bacterium, or if the gene is expressed in any particular habitat. The concept of HGT, where living bacteria can pick up genes released by dead bacteria, is a key element in the model describing how antibiotic resistance genes from animals can cause clinical health problems for humans. Knowing which bacteria are likely carrying specific antibiotic resistance genes raises the question of whether some bacteria are more relevant than others in the discussion of antibiotic resistance in food animals. For example, it has long been known that the major source of bacterial contamination on beef during processing comes from the animal hide, not the feces [26], and more recently this trend has been confirmed for pathogens such as Shiga-toxigenic *Escherichia coli* [27]. Results from the current study show that fluoroquinolone resistance in cattle feces is carried by both Bacteroidetes and Clostridia. Since Bacteroidetes are Gram-negative obligate anaerobes that don’t survive well outside of the animal gut and Clostridia are spore formers that can survive harsh environmental conditions found in the feedlot and on the hide, do the fluoroquinolone resistance genes in Clostridia have greater potential for transmission of antibiotic resistance to microorganisms important to humans? Across the 16 metagenomes for which taxonomic information was analyzed, the Bacteroidetes are associated with 24% of all fluoroquinolone resistance genes, compared to 15% associated with Clostridia. Another example, looking at beta-lactamase genes, is that while the Alpha- and Gamma-proteobacteria are most frequently associated with beta-lactamase resistance genes across metagenomes, in cattle feces, it is the Actinobacteria and Bacteroidetes bacteria that are most commonly linked with these genes – again raising the question of whether some bacteria are more relevant than others when considering the impacts of veterinary antibiotic use on human health. An additional factor which must be considered is the rate at which specific antibiotic resistance genes are transferred in feces and the environment, and the rate at which the modifications needed for expression of the transferred genes occurs. In order for the new host bacteria to become resistant the new host must have or acquire the genetic machinery needed to express the gene. Are some groups of bacteria more likely to acquire or contribute antibiotic resistance genes via HGT, and is the rate of HGT influenced by bacterial taxonomy and specific habitats? How do HGT rates as measured in the laboratory compare to actual rates in pre-harvest feedlot, food processing and storage, and environmental conditions? Since the taxonomic distribution of bacteria is different in each step of the farm-to-fork continuum [28] the dynamics of HGT may vary in each of these habitats. Knowing which bacteria are likely carrying specific antibiotic resistance genes affords a starting point for laboratory and field based experiments exploring HGT in food production settings.

Our results support those of Wright et al. [29] describing a global pool of antibiotic resistance genes. Studies showing that antibiotic resistance is ancient [30], when combined with work showing that antibiotic resistance genes can be found in a variety of human-impacted and pristine habitats ([29], [31], this study) reveal that the presence of antibiotic resistant genes is a normal and natural phenomenon. This means that, when looking at the impacts of veterinary use of antibiotics on human health, baseline studies and control samples are needed to determine natural background levels of antibiotic resistant bacteria and/or antibiotic resistance genes for comparison. By figuring out how antibiotic resistant bacteria and antibiotic resistance genes in agriculturally-impacted and non-agriculturally impacted environments compare, we can target control measures where they make the most sense.

### Conclusions

Antibiotic resistance genes are common in both agricultural and non-agricultural habitats, although differences exist in the diversity and taxonomic distribution of the bacteria associated with these genes in agricultural and non-agricultural metagenomes. Since antibiotic resistance genes are a natural part of both human-impacted and pristine habitats, presence of these resistance genes in any specific habitat therefore is not sufficient to indicate or determine impact of anthropogenic antibiotic use.

## Supporting Information

Figure S1 **RATC assignments for each of 26 metagenomes. Percent of RATC genes in each metagenome that are assigned to each RATC class. The highest values are denoted in red, the lowest values in green.**
(PDF)

Table S1 Metagenome Statistics.
(DOC)

Table S2 Resistance classes and genes used in this study.
(DOC)

Table S3 Distribution of five categories of resistance genes across 16 metagenomes, listed as percent of total genes in that category for all 16 metagenomes.
(DOC)
