# Supplementary material for: Distribution and Quantification of Antibiotic Resistant Genes and Bacteria across Agricultural and Non-Agricultural Metagenomes
Source: PLoS One. 2012 Nov 2;7(11):e48325. doi: 10.1371/journal.pone.0048325 (PMC3487761; doi:10.1371/journal.pone.0048325)
Supplement: Figure S1 — RATC assignments for each of 26 metagenomes. Percent of RATC genes in each metagenome that are assigned to each RATC class. The highest values are denoted in red, the lowest values in green. (PDF) [file pone.0048325.s001.pdf]

Percent of RATC assignments for each habitat that are assigned to each RATC class (based on total number of RATC assignments for each habitat)

Metagenome	Acriflavin	Aminogly	Arsenic	Bacitracin	Beta-lactamase	Cobaltzinccadmium	Fosfomycin	Integron	Meth Resist Staph	MexA-B OprM	MexC-D OprJ	MexE-F OprN	MDR efflux pump	MDR in Campy	MDR Gram Pos	MDR Gram Neg	MAR locus	Murcuric reductase	Murcury resist	Fluoroquinolone	Vancomycin	S pneumo Van	Streptothricin	Teicoplanin	Tetracycline	The mdtABC	Tolerance colicin	USS-db1	USS-db2	USS-db4	USS-db5	USS-db6	Zinc	sum of percent	%Virulence that is RATC	% RATC (of tot classifed seqs)	number RATC	Total # RATC classes
**Canine**	13.27	0.01	0.23	1.77	4.10	16.33	0.00	1.16	0.86	0.03	0.02	0.02	40.11	0.02	0.28	2.36	0.20	0.02	0.01	12.94	0.77	0.11	0.00	0.15	1.97	0.05	0.57	0.11	0.12	0.20	0.07	0.27	2.08	100	58	4.04	12286	31
**Soy phylosphere**	14.96	0.00	3.54	0.19	9.26	27.17	0.00	0.42	1.52	0.08	0.00	0.30	21.11	0.04	0.34	6.03	0.15	0.60	0.01	5.48	0.05	0.01	0.01	0.11	0.29	0.69	1.19	1.48	0.26	0.03	0.37	1.95	2.61	100	38	3.45	7366	30
**Human stool**	13.85	0.00	0.46	0.51	6.95	18.24	0.00	2.20	0.56	0.00	0.00	0.15	34.70	0.05	0.61	3.07	0.56	0.26	0.00	8.89	0.87	0.46	0.05	0.61	1.64	0.61	0.87	0.41	0.05	0.00	0.15	0.31	2.91	100	50	3.93	1957	27
**Cattle fecal**	6.81	0.11	0.16	0.93	6.31	14.09	0.00	1.45	0.77	0.16	0.00	0.00	39.54	0.05	0.61	1.51	0.00	0.00	0.25	19.96	0.63	0.05	0.00	0.14	5.06	0.07	0.11	0.07	0.14	0.02	0.05	0.07	0.09	99	44	3.75	4423	27
**Farm soil**	12.46	0.15	1.50	2.49	14.22	19.33	0.00	0.84	0.95	0.00	0.00	0.22	24.82	0.04	0.40	2.96	0.22	1.06	0.15	5.85	0.26	0.00	0.11	0.00	0.07	0.84	0.99	1.39	0.44	0.15	0.29	0.55	0.00	93	42	3.09	2736	27
**Chesapeake Bay**	13.91	0.00	0.64	0.48	9.88	23.47	0.00	0.27	1.07	0.00	0.00	0.16	15.25	0.05	0.54	2.85	0.21	0.21	0.00	23.74	0.27	0.11	0.00	0.11	2.58	0.11	0.48	0.59	1.34	0.00	0.00	0.21	1.45	100	44	2.00	1862	25
**Human F1-S**	8.21	0.13	0.64	2.56	3.33	9.36	0.00	0.90	1.50	0.00	0.00	0.00	48.46	0.13	0.38	1.92	0.13	0.13	0.00	12.56	1.54	0.38	0.00	0.26	3.85	0.13	0.00	0.77	0.64	0.00	0.00	0.26	2.18	100	46	3.81	780	24
**Whale fall**	16.39	0.00	0.77	0.99	12.21	34.54	0.00	0.88	0.77	0.00	0.00	0.44	16.28	0.11	0.11	2.97	0.44	0.77	0.11	4.51	0.00	0.00	0.00	0.00	0.22	0.11	0.55	0.22	0.11	0.00	0.33	1.54	4.62	100	41	3.60	909	24
**Zanzibar**	19.33	0.00	0.13	0.39	16.83	17.28	0.00	0.06	0.96	0.00	0.00	0.13	17.98	0.00	0.13	0.83	0.19	0.19	0.06	22.29	0.19	0.00	0.00	0.26	0.06	0.06	0.51	0.32	0.00	0.00	0.13	0.26	1.41	100	38	1.81	1557	24
**Key West**	17.37	0.00	0.90	0.36	15.62	17.25	0.06	0.12	0.60	0.00	0.00	0.24	18.46	0.00	0.30	1.33	0.12	0.06	0.06	24.00	0.00	0.00	0.06	0.36	0.06	0.18	0.12	0.42	0.00	0.00	0.00	0.12	1.81	100	38	1.70	1658	24
**Galapagos**	20.30	0.00	0.06	0.41	17.21	16.77	0.00	0.10	0.80	0.00	0.00	0.13	17.44	0.00	0.16	0.70	0.10	0.29	0.03	21.67	0.25	0.06	0.00	0.19	0.13	0.00	0.19	0.29	0.06	0.00	0.00	0.29	2.39	100	36	1.84	3143	24
**Gulf of Mexico**	16.61	0.00	0.72	0.30	17.81	16.13	0.00	0.12	0.66	0.00	0.00	0.00	18.17	0.00	0.90	1.92	0.24	0.06	0.00	21.58	0.24	0.06	0.06	0.12	0.24	0.12	0.30	0.30	0.12	0.00	0.00	0.18	3.06	100	36	1.78	1668	24
**Rumen planktonic**	2.60	0.20	0.40	1.20	4.90	6.29	0.00	0.00	0.40	0.00	0.00	0.20	31.67	0.00	0.00	0.20	0.00	0.20	0.00	30.67	2.60	0.10	0.10	0.00	16.88	0.20	0.30	0.10	0.00	0.00	0.10	0.00	0.70	100	64	2.88	1001	21
**Human InvA**	11.73	0.00	0.37	4.66	4.28	13.97	0.00	2.05	0.56	0.00	0.00	0.00	40.41	0.00	0.93	1.86	0.19	0.37	0.00	10.43	1.49	0.19	0.00	0.56	2.61	0.00	0.37	1.12	0.00	0.00	0.00	0.19	1.68	100	50	3.97	537	21
**Termite**	14.37	0.00	0.20	0.61	6.48	17.00	0.00	1.11	1.52	0.00	0.00	0.00	31.38	0.00	0.20	0.30	0.20	0.00	0.00	17.21	0.20	0.00	0.20	0.00	0.00	0.00	1.42	0.51	0.10	0.10	0.10	1.82	4.96	100	37	2.66	988	21
**Chicken A**	6.72	0.06	1.10	2.04	2.81	9.97	0.00	0.11	0.11	0.00	0.00	0.00	39.45	0.00	0.22	0.17	0.00	0.00	0.00	22.59	1.60	0.39	0.17	0.00	10.96	0.00	0.77	0.11	0.06	0.00	0.00	0.00	0.61	100	65	3.03	1815	20
**Rumen640F6**	1.85	0.19	0.58	1.55	4.08	7.97	0.00	0.19	0.39	0.00	0.00	0.00	28.86	0.00	0.00	0.10	0.00	0.00	0.00	29.74	1.46	0.10	0.00	0.00	21.67	0.00	0.19	0.29	0.19	0.00	0.00	0.10	0.49	100	65	2.92	1029	19
**Madagascar**	21.00	0.00	0.18	0.18	10.68	11.74	0.00	0.00	0.36	0.00	0.00	0.18	22.24	0.00	0.18	0.36	0.00	0.18	0.00	29.36	0.00	0.00	0.00	0.18	0.00	0.18	0.36	0.36	0.53	0.00	0.00	0.00	1.78	100	39	1.62	562	18
**Guerro Negro mat**	22.73	0.00	1.95	0.65	11.04	18.83	0.00	1.30	0.00	0.00	0.00	0.00	18.18	0.00	0.65	1.30	0.65	1.30	0.00	9.09	1.95	0.00	0.00	0.00	0.00	0.65	0.65	1.30	0.00	0.00	0.00	3.90	3.90	100	37	2.24	154	18
**Gulf of Maine**	20.84	0.00	0.00	0.16	17.46	20.76	0.00	0.16	1.37	0.00	0.00	0.16	16.73	0.00	0.72	0.80	0.08	0.00	0.00	17.62	0.00	0.00	0.00	0.16	0.00	0.08	0.24	0.00	0.00	0.00	0.08	0.48	2.09	100	31	1.57	1243	18
**Antarctic lake 1**	7.41	0.00	0.13	0.65	4.03	15.99	0.00	10.40	5.20	0.00	0.00	0.00	23.41	0.00	0.00	1.04	0.13	0.26	0.00	23.93	4.29	0.00	0.00	0.00	1.04	0.00	0.13	0.39	0.00	0.00	0.00	0.13	1.43	100	25	2.15	769	18
**Kimchi 29**	0.21	0.00	0.00	4.88	4.24	18.98	0.00	0.00	0.81	0.00	0.00	0.00	23.65	0.00	12.30	4.88	0.00	1.17	0.00	19.83	1.59	0.11	0.11	0.42	1.06	0.11	0.00	0.00	0.11	0.00	0.00	0.00	0.00	94	64	3.62	943	17
**Fish**	6.17	0.00	2.26	0.21	4.73	53.91	1.03	0.00	0.00	0.00	0.00	0.00	14.61	0.00	0.00	6.79	0.00	2.26	1.65	2.06	0.00	0.00	0.00	0.00	0.00	1.03	1.23	0.21	0.00	0.00	0.21	1.03	1.62	101	49	3.99	486	17
**Rumen80F6**	1.12	0.14	0.28	0.56	5.31	10.20	0.00	0.28	0.00	0.00	0.00	0.00	30.03	0.00	0.00	0.00	0.00	0.00	0.00	30.17	2.23	0.00	0.14	0.00	18.58	0.00	0.42	0.14	0.14	0.00	0.00	0.00	0.28	100	64	2.67	716	16
**Antartic lake 2**	10.47	0.00	0.00	2.33	3.49	20.93	0.00	8.14	6.98	0.00	0.00	0.00	9.30	0.00	0.00	0.00	1.16	0.00	0.00	22.09	6.98	0.00	0.00	0.00	1.16	0.00	0.00	3.49	0.00	0.00	0.00	2.33	1.16	100	32	2.24	86	14
**Sargasso**	28.95	0.00	2.63	0.00	2.63	5.26	0.00	0.00	2.63	0.00	0.00	0.00	18.42	0.00	0.00	0.00	0.00	0.00	0.00	39.47	0.00	0.00	0.00	0.00	0.00	0.00	0.00	0.00	0.00	0.00	0.00	0.00	0.00	100	24	0.70	38	7

KEY

Acriflavin	Acriflavinresistancecluster
Aminogly	Aminoglycoside-adenylyltransferases
Arsenic	Arsenic-resistance
Bacitracin	Bacitracin stress response
Beta-lactamase	Beta-lactamase
Cobaltzinccadmium	Cobaltzinccadmium
Fosfomycin	Fosfomycin resistance
Integron	Integron
Meth Resist Staph	Methicillin Resistance in Staphylococci
MexA-B OprM	MexA-MexB-OprM_Multidrug_Efflux_System
MexC-D OprJ	MexC-MexD-OprJ_MDR Multidrug_Efflux_Syste
MexE-F OprN	MexE-MexF-OprN_Multidrug_Efflux_System
MDR efflux pump	Multi Drug Resistance Efflux Pumps
MDR in Campy	Multidrug efflux pump in Campylobacter Jejuni
MDR Gram Pos	Multidrug_Resistance, 2-proten Gram Pos
MDR Gram Neg	Multidrug_Resistance, Tripartite Gram Neg
MAR locus	Multiple Antibiotic Resistance MAR locus
Murcuric reductase	Murcuric-reductase
Murcury resist	Murcury_reistance_operon
Fluoroquinolone	Resistance to Floroquinolones
Vancomycin	Resistance to Vancomycin
S pneumo Van	Streptococcus_pneumoniae_Van_Tolerance Lo
Streptothricin	Streptothricin resistance
Teicoplanin	Teicoplanin-resistance-in-Staphylococcus
Tetracycline	Tetracycline_resistance, ribosome protection ty
The mdtABC	The mdtABC multidrug resistance cluster
Tolerance colicin	Tolerance to coloicin E2
USS-db1	USS-db1
USS-db2	USS-db2
USS-db4	USS-db4
USS-db5	USS-db5
USS-db6	USS-db6
Zinc	zincresistance
