# Supplementary material for: Distribution and Quantification of Antibiotic Resistant Genes and Bacteria across Agricultural and Non-Agricultural Metagenomes
Source: PLoS One. 2012 Nov 2;7(11):e48325. doi: 10.1371/journal.pone.0048325 (PMC3487761; doi:10.1371/journal.pone.0048325)
Supplement: Table S1 — Metagenome Statistics. (DOC) [file pone.0048325.s002.doc]

**Supplementary Table 1. Metagenome Statistics.**

| **ID #** | **habitat** | **Type** | **MGRASTID** | **Total # of Sequence in Metagenome** | **Total # Classified sequences** | **# of Sequences categorized as "virulence"** | **# of virulence sequence that are RATC** | **% of RATC (based on tot classified)** | **Lat** | **Long** | **Source** | **Citation** |
| --- | --- | --- | --- | --- | --- | --- | --- | --- | --- | --- | --- | --- |
| 1 | Antarctic lake 1 | Fresh water | 4443683.3 | 100,085 | 35764 | 3020 | 769 | 2.15 | -68.4 | 78.18278 | JM Hoffman | Ng et al. 2010 |
| 2 | Antarctic lake 2 | Fresh water | 4443680.3 | 9,672 | 3842 | 266 | 86 | 2.24 | -68.4 | 78.18278 | JM Hoffman | Ng et al. 2010 |
| 3 | Sargasso Sea | Sea water | 441624.3 | 399,343 | 5434 | 160 | 38 | 0.70 | 32.1667 | -64.5 | F Rohwer | Angly et al. 2006 |
| 4 | Galapagos costal water | Sea water | 4441595.3 | 222,080 | 171241 | 8656 | 3143 | 1.84 | 1.2161 | -90.4228 | JC Venter | Rusch et al. 2007 |
| 5 | Cattle fecal | Feces | 4448367.3 | 273,960 | 118007 | 9900 | 4423 | 3.75 | 40.52168 | -98.0553 | LM Durso | Durso et al. 2011 |
| 6 | Rumen 80F6 | Rumen | 4441680.3 | 178,713 | 26861 | 1119 | 716 | 2.67 | 40.5109 | -88.9916 | B White | Brulc et al. 2009 |
| 7 | Rumen planktonic | Rumen | 4441682.3 | 236,830 | 34735 | 1549 | 1001 | 2.88 | 40.5109 | -88.9916 | B White | Brulc et al. 2009 |
| 8 | Chicken cecum A | Feces | 4440283.3 | 294,682 | 59850 | 2771 | 1815 | 3.03 | 40.1106 | -88.2073 | B White | Qu et al. 2008 |
| 9 | Gulf of Maine | Sea water | 441579.3 | 121,590 | 79364 | 3895 | 1243 | 1.57 | 42.5031 | -67.24 | JC Venter | Rusch et al. 2007 |
| 10 | Chesapeake Bay | Sea water | 4441584.3 | 126,162 | 92970 | 4192 | 1862 | 2.00 | 38.9469 | -76.4172 | JC Venter | Rusch et al. 2007 |
| 11 | Key West | Sea water | 4441586.3 | 127,362 | 97784 | 4354 | 1658 | 1.70 | 24.4883 | -83.07 | JC Venter | Rusch et al. 2007 |
| 12 | Guerrero Negro 0-1mm | Microbial mat | 4440964.3 | 12,213 | 6889 | 418 | 154 | 2.24 | 27.68908 | -113.917 | P Hugenholtz | Kunin et al 2008 |
| 13 | Human In-A feces | Feces | 4440946.3 | 20,226 | 13527 | 1059 | 537 | 3.97 | 35.7134 | 139.7623 | M Hattor | Kurokawa et al 2007 |
| 14 | Canine | Feces | 4444702.3 | 583,523 | 304042 | 20816 | 12286 | 4.04 | 40.09304 | -88.221 | G Fahey Jr | Swanson et al. 2010 |
| 15 | Soy leaf | Plant | 4447793.3 | 504,525 | 213583 | 19189 | 7366 | 3.45 | no data | no data | no data | Not available |
| 16 | Kimchi d29 | Food | 4450218.3 | 48,813 | 26018 | 1421 | 943 | 3.62 | 37.56654 | 126.978 | CO Jeon | Jung et al 2011 |
| 17 | Fish | Feces | 4441695.3 | 51498 | 12192 | 993 | 486 | 3.99 | 33.537594 | -116.098 | F Angly | Dinsdale et al. 2008 |
| 18 | Whale fall mat | Microbial mat | 441656.4 | 38281 | 25232 | 2228 | 909 | 3.60 | 33.3 | -119.22 | EM Rubin | Tringe et al, 2005 |
| 19 | Termite gut | Feces | 4442701.3 | 57641 | 37144 | 2665 | 988 | 2.66 | 10.11 | -83.51 | JR Leadbetter | Warnecke et al, 2007 |
| 20 | Rumen 640F6 | Rumen | 4441679.3 | 264,849 | 35267 | 1561 | 1029 | 2.92 | 40.5109 | -88.9916 | B White | Brulc et al. 2009 |
| 21 | Human stool | Feces | 4444130.3 | 108,486 | 49749 | 3868 | 1957 | 3.93 | no data | no data | no data | Not available |
| 22 | Human F1-S | Feces | 4440939.3 | 28,900 | 20461 | 1659 | 780 | 3.81 | 35.7134 | 139.7623 | M Hattor | Kurokawa et al 2007 |
| 23 | Farm Soil | Soil | 4441091.3 | 138,347 | 88637 | 6444 | 2736 | 3.09 | 43.9614 | -93.6623 | EM Rubin | Tringe et al, 2005 |
| 24 | Madagascar | Sea water | 4441135.3 | 46,052 | 34771 | 1429 | 562 | 1.62 | -26.035 | 50.123 | JC Venter | Rusch et al. 2007 |
| 25 | Zanzibar | Sea water | 4441618.3 | 110,984 | 86257 | 4067 | 1557 | 1.81 | -6.1167 | 39.1167 | JC Venter | Rusch et al. 2007 |
| 26 | Gulf of Mexico | Sea water | 4441660.3 | 127,122 | 93695 | 4662 | 1668 | 1.78 | 24.1747 | -84.344 | JC Venter | Rusch et al. 2007 |
