# Supplementary material for: Distribution and Quantification of Antibiotic Resistant Genes and Bacteria across Agricultural and Non-Agricultural Metagenomes
Source: PLoS One. 2012 Nov 2;7(11):e48325. doi: 10.1371/journal.pone.0048325 (PMC3487761; doi:10.1371/journal.pone.0048325)
Supplement: Table S2 — Resistance classes and genes used in this study. (DOC) [file pone.0048325.s003.doc]

**Supplementary Table 2**. Resistance classes and genes used in this study.

**List of RATC classes found in 26 metagenomes**

Acriflavin resistance cluster

Aminoglycoside-adenylyltransferases

Arsenic-resistance

Bacitracin stress response

Beta-lactamase

Cobalt zinc cadmium resistance

Fosfomycin resistance

Integron

Methicillin Resistance in Staphylococci

MexA-MexB-OprM Multidrug Efflux System

MexC-MexD-OprJ Multidrug Efflux System

MexE-MexF-OprN Multidrug Efflux System

Multi Drug Resistance Efflux Pumps

Multidrug efflux pump in Campylobacter jejuni

Multidrug Resistance, 2-proten Gram Positive

Multidrug Resistance, Tripartite Gram Negative

Multiple Antibiotic Resistance MAR locus

Meurcuric-reductase

Mercury resistance operon

Resistance to Floroquinolones

Resistance to Vancomycin

Streptococcus pneumoniae Van Tolerance Locus

Streptothricin resistance

Teicoplanin resistance in Staphylococcus

Tetracycline resistance, ribosome protection type

The mdtABC multidrug resistance cluster

Tolerance to colicin E2

USS-db1

USS-db2

USS-db4

USS-db5

USS-db6

Zinc resistance

**Beta-lactamase resistance sub-categories**

Beta-lactamase (Cephalosporinase) (EC 3.5.2.6)

Beta-lactamase (EC 3.5.2.6)

Beta-lactamase class A

Beta-lactamase class C and other penicillin binding proteins

Beta-lactamase class D

Beta-lactamase repressor BlaI

Metal-dependent hydrolases of the beta-lactamase superfamily I

Metal-dependent hydrolases of the beta-lactamase superfamily II

Metal-dependent hydrolases of the beta-lactamase superfamily III

Negative regulator of beta-lactamase expression

Probable beta-lactamase ybxI precursor (EC 3.5.2.6)

Regulatory protein blaR1

**MDR Efflux Pumps sub-categories**

Inner membrane efflux transporter of RND family multidrug efflux pump

Macrolide export ATP-binding/permease protein macB (EC 3.6.3.-)

Macrolide-specific efflux protein MacA

Membrane fusion protein of RND family multidrug efflux pump

Multi antimicrobial extrusion protein (Na(+)/drug antiporter) MATE family of MDR efflux pumps

**Flurorquinolone resistance sub-categories**

DNA gyrase subunit A (EC 5.99.1.3)

DNA gyrase subunit B (EC 5.99.1.3)

Efflux pump Lde

Topoisomerase IV subunit A (EC 5.99.1.-)

Topoisomerase IV subunit B (EC 5.99.1.-)

**Tetracylcine resistance sub-categories**

Oxytetracycline resistance protein OtrA

Ribosome protection-type tetracycline resistance related proteins

Ribosome protection-type tetracycline resistance related proteins, group 2

Tetracycline resistance protein TetM

Tetracycline resistance protein TetO

Tetracycline resistance protein TetP

Tetracycline resistance protein TetQ

Tetracycline resistance protein TetW

Translation elongation factor G

**Vancomycin resistance sub-categories**

Sensor histidine kinase VanS (EC 2.7.3.-)

Vancomycin B-type resistance protein vanW

Vancomycin B-type resistance protein VanX

Vancomycin resistance protein VanH

Vancomycin response regulator VanR

Sensor histidine kinase VanS (EC 2.7.3.-)

Vancomycin resistance protein VanH

Vancomycin response regulator VanR
