# Supplementary material for: Distribution and Quantification of Antibiotic Resistant Genes and Bacteria across Agricultural and Non-Agricultural Metagenomes
Source: PLoS One. 2012 Nov 2;7(11):e48325. doi: 10.1371/journal.pone.0048325 (PMC3487761; doi:10.1371/journal.pone.0048325)
Supplement: Table S3 — Distribution of five categories of resistance genes across 16 metagenomes, listed as percent of total genes in that category for all 16 metagenomes. (DOC) [file pone.0048325.s004.doc]

**Supplementary Table 3. Distribution of five categories of resistance genes across 16 metagenomes, listed as percent of total genes in that category for all 16 metagenomes.**

| **Metagenome** | **Betalactamase (n=2,919)** | **MDR Efflux**  **(n= 10,884)** | **Fluoroquinolone**  **(n=6,025)** | **Tetracycline (n=1084)** | **Vancomycin (n=275)** |
| --- | --- | --- | --- | --- | --- |
| Human | 0.8 | 1.6 | 3.1 | 1.3 | 2.9 |
| Canine | 17.3 | 45.9 | 0.3 | 22.2 | 34.1 |
| Cattle Fecal | 9.6 | 16.2 | 26.4 | 20.7 | 10.2 |
| Chicken cecum | 1.7 | 0.5 | 14.7 | 18.4 | 10.5 |
| Rumen 80F6 | 1.3 | 2.0 | 7.4 | 12.3 | 5.8 |
| Rumen Plank | 1.7 | 2.9 | 6.8 | 15.6 | 9.5 |
| Gulf of Maine | 7.4 | 1.9 | 3.6 | 0.0 | 0.0 |
| Chesapeak | 6.3 | 2.6 | 11.3 | 4.4 | 1.8 |
| Key West | 8.9 | 2.8 | 0.2 | 0.1 | 0.0 |
| Galapagos | 18.4 | 5.0 | 0.9 | 0.4 | 2.9 |
| Sargasso | 0.0 | 0.1 | 6.6 | 0.0 | 0.0 |
| Antarctic 1 | 1.1 | 1.6 | 3.1 | 0.7 | 12.0 |
| Antarctic 2 | 0.1 | 0.1 | 3.6 | 0.1 | 2.2 |
| Guerro mat | 0.6 | 0.3 | 5.1 | 0.0 | 1.1 |
| Soy leaf | 23.4 | 14.5 | 0.2 | 2.9 | 1.5 |
| Kimchi | 1.4 | 2.0 | 6.7 | 0.9 | 5.5 |
| **Total** | 100 | 100 | 100 | 100 | 100 |
